# Cone-Beam CT and Image Fusion-Guided Percutaneous Recanalization of Occluded Central Venous Stent

**DOI:** 10.1016/j.jaccas.2021.09.016

**Published:** 2021-12-01

**Authors:** Lamees I. El Nihum, M. Mujeeb Zubair, Ponraj Chinnadurai, Eric K. Peden

**Affiliations:** a Texas A&M College of Medicine, Bryan, Texas, USA; b Smidt Heart Institute, Cedars-Sinai Medical Center, Los Angeles, California, USA; c Advanced Therapies, Siemens Medical Solutions USA, Inc., Malvern, Pennsylvania, USA; d DeBakey Heart & Vascular Center, Houston Methodist Hospital, Houston, Texas, USA

**Keywords:** central venous occlusion, image fusion, sharp needle recanalization, BCV, brachiocephalic vein, CBCT, cone-beam computed tomography, CVO, central venous occlusion, DSA, digital subtraction angiography, ESRD, end-stage renal disease, IJV, internal jugular vein, LUE, left upper extremity, MRV, magnetic resonance venography, SCV, subclavian vein, SVC, superior vena cava, TDC, tunneled dialysis catheter

## Abstract

We describe an 81-year-old man with end-stage renal disease and central venous occlusion who was referred for dialysis access creation. This case illustrates a novel percutaneous image fusion–guided recanalization of an occluded right subclavian vein and brachiocephalic vein stent in a patient with limited remaining dialysis access sites. (**Level of Difficulty: Advanced.**)

## Central Illustration

**Figure:**
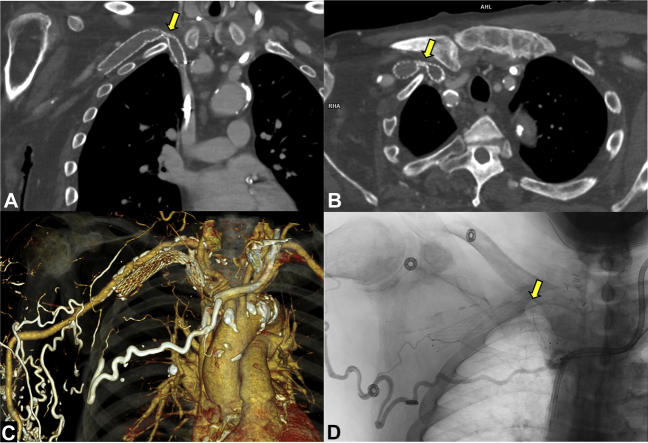

## History of Presentation

An 81-year-old man with end-stage renal disease (ESRD), central venous occlusion (CVO), and chronic left upper extremity (LUE) lymphedema presented for evaluation of dialysis access creation.

**Learning Objectives**

• To demonstrate an image-guided approach to recanalize an occluded central venous stent in a patient with no available access sites for dialysis.
• To illustrate how intraoperative cross-sectional CBCT imaging with image fusion may improve the safety of such percutaneous recanalization approaches when conventional endovascular approaches fail.
• To emphasize that image-guided approaches may facilitate novel percutaneous (outside-in) or inside-out access strategies that are evolving for recanalizing central venous occlusion.

## Past Medical History

The patient had a history of ESRD with hemodialysis through a left internal jugular vein (IJV) tunneled dialysis catheter (TDC) and chronic LUE lymphedema. His previous history included a kidney transplant, right arm fistulas, and multiple dialysis catheters. He also had a previously placed stent in the right subclavian vein (SCV) extending into the right brachiocephalic vein (BCV).

## Investigations

Bilateral upper extremity venograms showed severe stenosis of the left BCV at the site of TDC, as well as a right occluded SCV-BCV stent with extensive chest wall collateral vessels.

**Figure 1:**
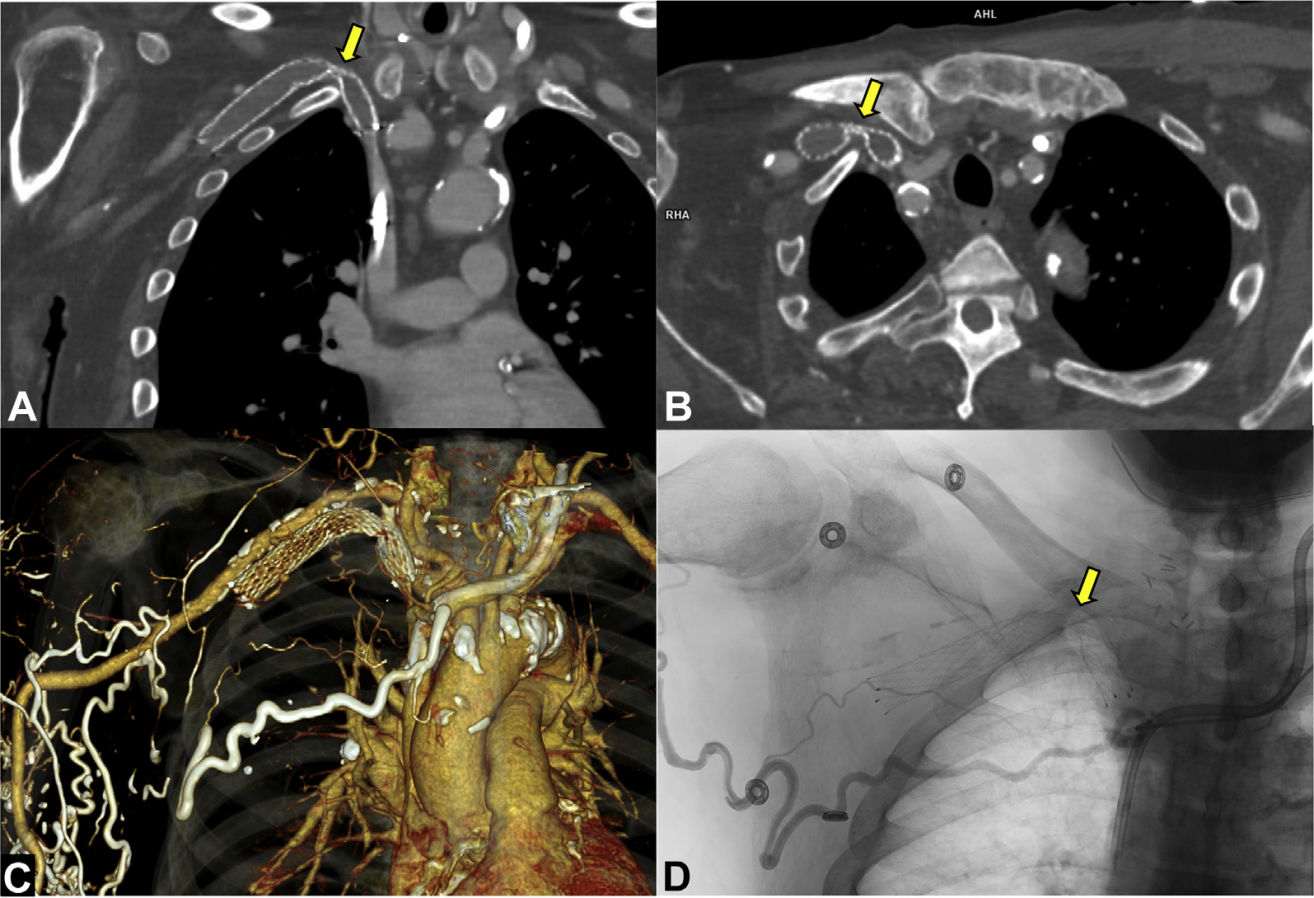
Computed Tomography Venography Demonstrating an Occluded Right Subclavian Vein and Brachiocephalic Vein Stent **(A)** Coronal, **(B)** axial, and **(C)** 3-dimensional volume-rendered reconstruction of computed tomography venography demonstrated no contrast opacification inside the stent. The stent was crushed between the posteromedial end of the clavicle and the first rib **(yellow arrows in A and B). (D)** Right arm venography showed a deformed, occluded stent **(yellow arrow)** and collateral vessels.

Computed tomography (CT) angiography demonstrated an occluded right-sided stent maximally narrow between the clavicle and the first rib (Figures 1A to 1D), thus suggesting thoracic outlet–type compression of the central SCV.

## Management

Various treatment options were considered, including a chest wall graft (left chest wall arteriovenous graft placement with stenting of the left BCV stenosis) and a lower extremity arteriovenous fistula or graft.

We elected to proceed with intraoperative cone-beam CT (CBCT) and image fusion–guided percutaneous recanalization of the occluded right SCV-BCV stent with central venous catheter placement on the right side, to be later converted to a right arm HeRO Graft (Merit Medical Systems), a hybrid combination graft-catheter implant for arteriovenous access.

**Figure 2:**
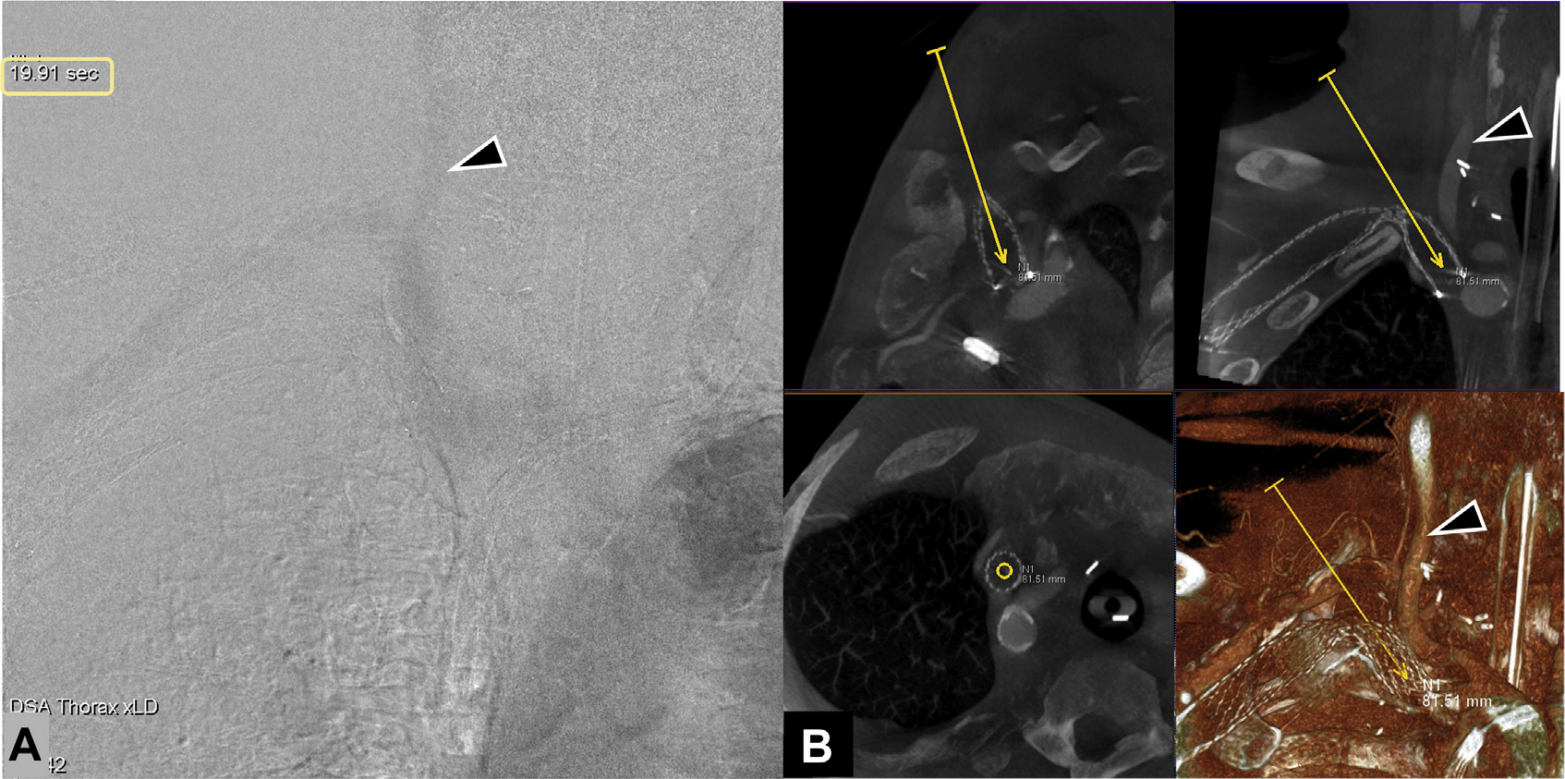
Timing Bolus Digital Subtraction Angiography and Intraoperative Cone-Beam Computed Tomography Venography Planning **(A)** Timing bolus digital subtraction angiography was performed to derive the time to visualize arterial structures such as the right subclavian artery and the right common carotid artery **(arrowhead)** before cone-beam computed tomography angiography. **(B)** A virtual needle path (N1) was planned **(yellow line)** in 3-dimensional multiplanar and volume-rendered reconstructions of cone-beam computed tomography images. The **yellow circle** in the axial view denotes the target site inside the occluded stent. The **arrowhead** denotes the right common carotid artery. N1 = needle path 1.

Right femoral venous access was obtained, and a 5-F sheath was inserted. To derive the timing of CBCT image acquisition to visualize arterial vasculature, timing bolus digital subtraction angiography (DSA) imaging of the chest was performed after injecting 24 mL of an iodinated contrast agent (iohexol [Omnipaque], 300 mg/mL) at 8 mL/s for 3 seconds through the femoral venous sheath by using a power injector. The aortic arch and great vessels were visualized in DSA imaging approximately 20 seconds after the start of the contrast medium injection (Figure 2A). Intraoperative CBCT acquisition was performed, timed for arterial enhancement of the chest and lower neck, 20 seconds (x-ray delay) after injecting 40 mL of an iodinated contrast agent at 8 mL/s using a 5-second scan protocol (DynaCT, Siemens Healthineers) in a hybrid operating room equipped with a robotic-assisted angiography system (ARTIS pheno VE10, Siemens Healthineers). A virtual needle path was planned using dedicated software (syngo Needle Guidance, Siemens Healthineers) in multiplanar reconstructions of CBCT extending from the planned skin access site in the right side of the neck and traversing through the interstices into the occluded stent toward the patent superior vena cava (SVC) (Figure 2B). Image fusion guidance allowed for planning puncture of the more vertical portion of the occluded stent medial to the crushed portion in the area of compression from the first rib and the clavicle. The lateral margin of the right common carotid artery and the superior margin of the right subclavian artery were marked in CBCT and overlaid on live fluoroscopy to avoid arterial injury.

**Figure 3:**
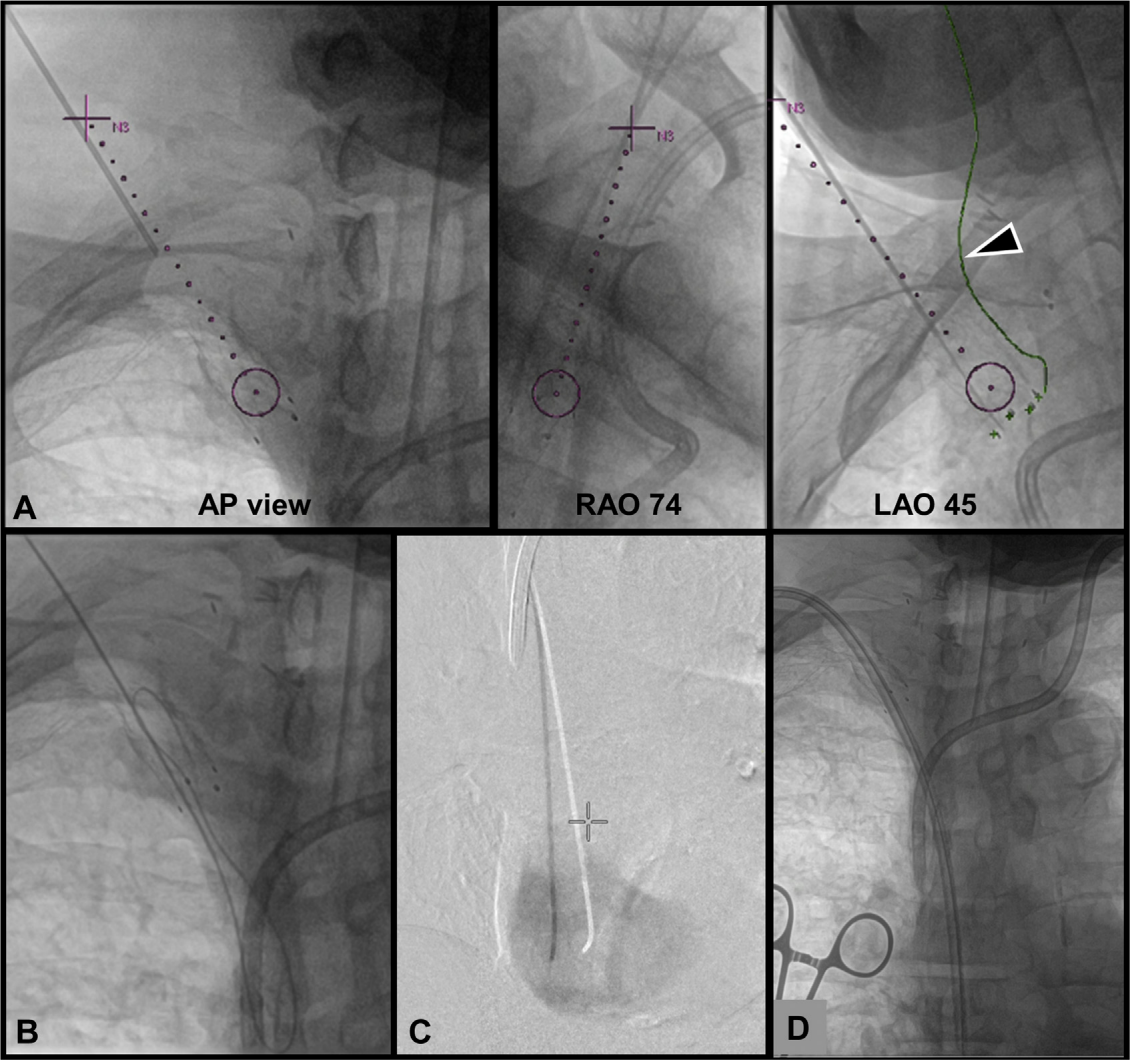
Intraoperative Cone-Beam Computed Tomography and Fluoroscopy Image Guidance for Percutaneous Recanalization of the Occluded Stent **(A)** Overlay of a 3-dimensional virtual path from cone-beam computed tomography onto 2-dimensional fluoroscopy for guiding percutaneous recanalization of an occluded stent **(purple dotted line); the green line** indicates the lateral margin of the right common carotid artery. **(B)** Puncture and wire passage into the superior vena cava (SVC) with **(C)** confirmation of catheter position in the right atrium. **(D)** Placement of a tunneled dialysis catheter. AP = anteroposterior; LAO = left anterior oblique; RAO = right anterior oblique.

**Figure 4:**
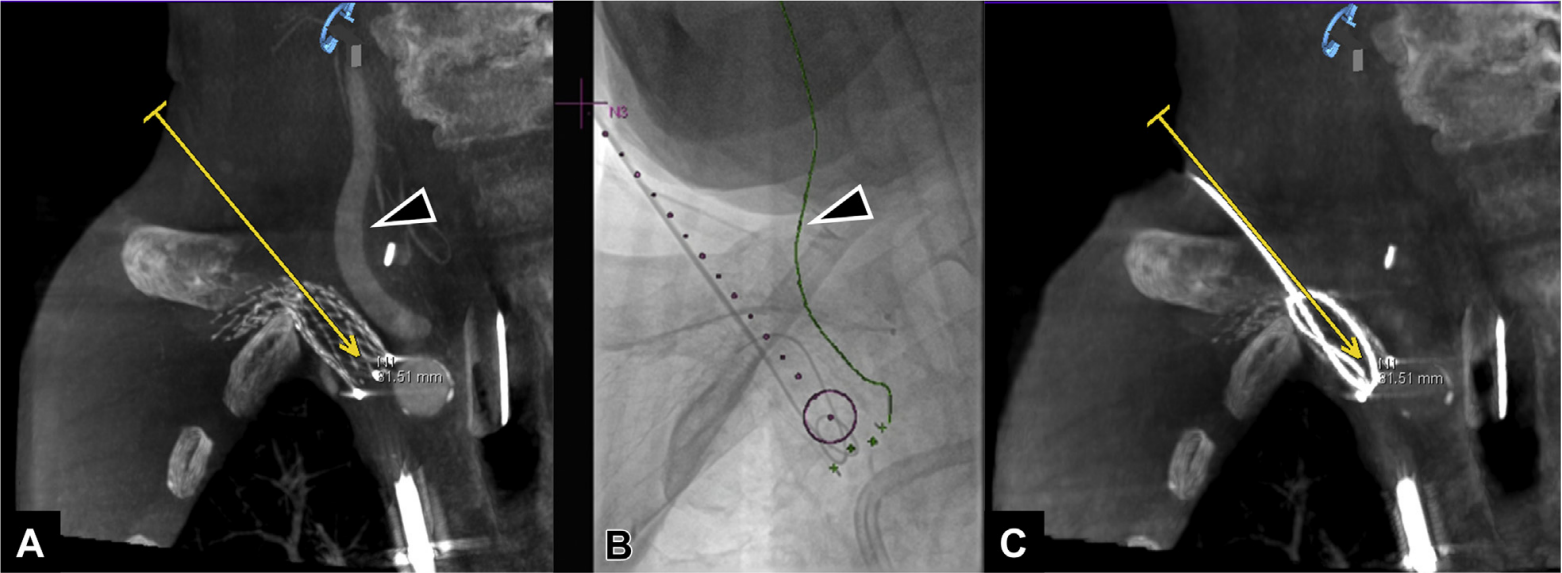
Cone-Beam Computed Tomography Before and After Percutaneous Recanalization of the Occluded Stent **(A)** Coronal reconstruction of intraoperative cone-beam computed tomography images, showing the planned virtual needle path **(yellow line)** and its relationship to the right subclavian artery and the right common carotid artery **(arrowhead)**. **(B)** Insertion of an 18-gauge needle along the virtual needle path from cone-beam computed tomography overlaid on 2-dimensional fluoroscopy **(purple dotted line)**. The **purple circle** indicates the target site inside the occluded stent. The **green line** indicates the lateral margin of the right common carotid artery (denoted by the **arrowhead**). **(C)** Coronal reconstruction of postrecanalization noncontrast cone-beam computed tomography confirming the location of the guidewire inside the occluded stent along the planned virtual needle path **(yellow line)**. N1 = needle path 1; N3 = needle path 3.

Percutaneous needle access to the occluded right SCV-BCV stent was then performed under fluoroscopic guidance at multiple C-arm angulations overlaid with the virtual needle path from CBCT (Figure 3A). An 18-gauge needle was used to puncture the skin above the medial clavicle to enter the occluded stent in the right BCV. A stiff glidewire (Terumo Medical Canada, Inc) was advanced into the inferior vena cava (Figure 3B). The occluded stent was successfully recanalized, and a catheter was positioned in the right atrium with placement confirmed by angiography (Figures 3C and 4A to 4C).

A tunnel was then created on the right anterior chest wall with a counterincision. We passed a 6-F sheath over the stiff wire and performed angioplasty of the stent with a 6-mm balloon to make room for the dialysis catheter. After measuring the appropriate length under fluoroscopic guidance, a 27-cm TDC was tunneled from the lower counterincision and placed through the occluded stent into the right atrium (Figure 3D). This TDC was secured to the chest wall, and the left IJV TDC was removed.

## Discussion

CVO in patients with ESRD can present a life-threatening situation. To continue with dialysis in the setting of CVO, various treatment modalities can be considered (1). For most lesions, standard wire and catheter techniques will suffice for crossing. Alternatives include sharp needle recanalization, in which the occluded venous segment is traversed with a needle under image guidance, or use of a wire with radiofrequency energy to cross (2). Subsequent interventions in the event of failure of recanalization include surgical reconstruction of the central veins, all arterial access that does not involve venous outflow, lower extremity access, or conversion to peritoneal dialysis. Most recently, the Surfacer Inside-Out Access Catheter System (Merit Medical Systems) has been approved to offer a new option to patients with CVO.

We previously described magnetic resonance venography (MRV) and CBCT image fusion guidance for endovascular recanalization of chronic CVO, in which patients with failed standard endovascular recanalization underwent successful recanalization when aligning virtual centerlines from MRV with an actual guidewire trajectory on fluoroscopy (3). In the current case, we used image fusion guidance to facilitate a percutaneous approach of a hemodialysis catheter placed as a bridge to a future hybrid combination graft-catheter implant for arteriovenous access. Outside-in sharp needle recanalization was performed under image fusion guidance. Fusion of intraoperative CBCT with fluoroscopy provided visualization of vital structures (carotid artery, subclavian artery, and trachea) and allowed for a clear needle trajectory into an occluded central venous stent medial to a crushed segment from thoracic outlet compression. We believe that our novel technique can allow for safer and more directed recanalization of occluded venous segments than achievable with standard guidewire recanalization.

Wellons et al (4) reported a similar technique of a fluoroscopically guided supraclavicular approach for transthoracic SVC permanent hemodialysis catheter placement in patients with bilateral IJV and SCV occlusion, without image fusion. In the described technique, a diagnostic catheter was inserted into the SVC from the femoral approach, and a venogram was obtained to assess the SVC location in relation to the right clavicle (4). The catheter was left in place for guidance during needle passage (4). Right supraclavicular access directly into the SVC was performed using a standard 18-gauge 2.75-inch introducer needle, using the anterior-posterior venogram as a reference guide (4). Once needle access was gained to the SVC, a wire was passed, and catheter placement proceeded in routine fashion (4). Of the 22 patients studied, 2 had major complications, including pneumothorax and hemothorax, and were successfully treated with chest tube decompression (4). With our described technique, both the pleural space and the major vascular structures can be visualized and avoided during puncture.

## Follow-Up

At 1 month, the patient presented for conversion of his TDC to a hybrid combination graft-catheter implant for arteriovenous access. The patient tolerated the procedure well and was discharged the same day.

## Conclusions

CBCT and image fusion guidance were used to treat a patient with ESRD who presented with limited remaining access sites for dialysis. This case demonstrates the added value of intraoperative 3D cross-sectional imaging with fusion to facilitate a percutaneous, image-guided approach to venous recanalization in patients with severe CVO.

## Funding Support and Author Disclosures

Dr Chinnadurai is a full-time employee and Senior Key Expert at Advanced Therapies Division, Siemens Medical Solutions USA, Inc. Dr Peden has served as a consultant for TVA Medical/Bard/BD Medical, Merit Medical, Humacyte, Inc., Venostent, Merck Sharpe & Dohme Corp., Laminate Medical Technologies, LTD, and Artegraft, Inc.; has served as a study quality committee member for Humacyte, Inc., Merck Sharpe & Dohme Corp., and Artegraft, Inc.; has participated in training programs for BD Medical and Merit Medical; has contracted research with Venostent; and has elected that all funds are retained by Houston Methodist Hospital. All other authors have reported that they have no relationships relevant to the contents of this paper to disclose.
